# Evaluation of Clinical Risk Factors to Predict High On-Treatment Platelet Reactivity and Outcome in Patients with Stable Coronary Artery Disease (PREDICT-STABLE)

**DOI:** 10.1371/journal.pone.0121620

**Published:** 2015-03-23

**Authors:** Michal Droppa, Dimitri Tschernow, Karin A. L. Müller, Elli Tavlaki, Athanasios Karathanos, Fabian Stimpfle, Elke Schaeffeler, Matthias Schwab, Alexander Tolios, Jolanta M. Siller-Matula, Meinrad Gawaz, Tobias Geisler

**Affiliations:** 1 Department of Cardiology and Cardiovascular Medicine, University Hospital of Tübingen, Tübingen, Germany; 2 Dr. Margarete Fischer-Bosch-Institute of Clinical Pharmacology, Stuttgart, Germany; 3 Department of Clinical Pharmacology, University Hospital of Tübingen, Tübingen, Germany; 4 Laboratory Medicine Institute, Medical Center of the University of Munich, Munich, Germany; 5 Department of Cardiology, Medical University of Vienna, Vienna, Austria; Medizinische Hochschule Hannover, GERMANY

## Abstract

**Objectives:**

This study was designed to identify the multivariate effect of clinical risk factors on high on-treatment platelet reactivity (HPR) and 12 months major adverse events (MACE) under treatment with aspirin and clopidogrel in patients undergoing non-urgent percutaneous coronary intervention (PCI).

**Methods:**

739 consecutive patients with stable coronary artery disease (CAD) undergoing PCI were recruited. On-treatment platelet aggregation was tested by light transmittance aggregometry. Clinical risk factors and MACE during one-year follow-up were recorded. An independent population of 591 patients served as validation cohort.

**Results:**

Degree of on-treatment platelet aggregation was influenced by different clinical risk factors. In multivariate regression analysis older age, diabetes mellitus, elevated BMI, renal function and left ventricular ejection fraction were independent predictors of HPR. After weighing these variables according to their estimates in multivariate regression model, we developed a score to predict HPR in stable CAD patients undergoing elective PCI (PREDICT-STABLE Score, ranging 0-9). Patients with a high score were significantly more likely to develop MACE within one year of follow-up, 3.4% (score 0-3), 6.3% (score 4-6) and 10.3% (score 7-9); odds ratio 3.23, P=0.02 for score 7-9 vs. 0-3. This association was confirmed in the validation cohort.

**Conclusions:**

Variability of on-treatment platelet function and associated outcome is mainly influenced by clinical risk variables. Identification of high risk patients (e.g. with high PREDICT-STABLE score) might help to identify risk groups that benefit from more intensified antiplatelet regimen. Additional clinical risk factor assessment rather than isolated platelet function-guided approaches should be investigated in future to evaluate personalized antiplatelet therapy in stable CAD-patients.

## Introduction

Guidelines currently recommend dual platelet inhibition with aspirin and clopidogrel to prevent post-procedural adverse events after elective percutaneous coronary intervention (PCI) with stent-implantation [1]. There has been cumulative evidence in the past that interindividual variability of response to clopidogrel is high, mainly due to clinical risk factors and genetic variability of drug-metabolism [2–4]. There has been consensus that high-on-treatment platelet reactivity is associated with major adverse atherothrombotic events including stent thrombosis after PCI [5]. Presently new drugs with higher platelet inhibition and lower interindividual variability are available in clinical practice, however are not approved in stable coronary artery disease (CAD). Intensified platelet inhibition solely guided by platelet function analysis has been shown unsuccessful in reducing cardiovascular risk [6–8]. Thus, additional risk assessment is needed to identify patients with stable CAD who might benefit from enhanced platelet inhibition in the chronic phase.

Previously, we established a simple risk tool—PREDICT (Residual **P**latelet Agg**re**gation after **D**eployment of **I**ntra**c**oronary S**t**ent) score, based on clinical variables that are easily available in daily routine to identify patients at risk for high on-treatment platelet reactivity (HPR) in unselected cohort of patients undergoing PCI [9]. The score encompasses 5 different variables including acute coronary syndrome on admission, older age, diabetes mellitus, renal and left ventricular function impairment. After weighing these variables according to their effects size in multivariate analysis, the score ranged from 0–9 with higher score levels being significantly associated with both HPR and cardiovascular outcome. To date, tools to assess atherothrombotic risk after non-urgent PCI are lacking. Therefore, the aim of the present study was a) to identify clinical risk factors that are associated with on-treatment platelet reactivity and outcome and b) to investigate the added value of on-treatment platelet reactivity compared to clinical risk factor assessment in a selected population of patients with stable CAD undergoing PCI.

## Methods

### Study population

Patients with symptomatic coronary artery disease undergoing non-urgent coronary stent implantation were consecutively enrolled at the Department of Cardiology, University Hospital, Tübingen from March 2005 till May 2008. Inclusion criteria were age older than 18 years, planned coronary intervention and willing consent. Exclusion criteria were known platelet function disorders or indication for longterm oral anticoagulation. All patients were evaluated by platelet function analysis by Light Transmission Aggregometry (LTA) under maintenance therapy at a median of 24 hours after PCI. For the current analysis only patients with stable CAD were included and examined for clinical variables influencing HPR.

For the validation cohort, stable CAD patients with the same inclusion criteria presented between February 2011 and October 2012 at the University Hospital Tübingen, Germany (n = 354) and between March 2007 and September 2008 at the Department of Cardiology, Medical University of Vienna, Austria (n = 237) were analysed. The characteristics of the Austrian cohort are described elsewhere [10]. Platelet function was assessed using Multiple Electrode Aggretometry (MEA).

The trial was conducted in accordance with the principles of good clinical practice and the Declaration of Helsinki. All patients gave written informed consent. Approval was obtained by the ethical committee of the University Tübingen.

### Platelet function analysis

Platelet function analysis by Light Transmission Aggregometry (LTA) was performed at a median of 24 hours after a 600mg loading dose of clopidogrel was given. According to previous results, maximum platelet inhibition can be detected at this time point [9,11,12]. Venous blood samples collected in 3.8% citrate plasma were centrifuged at 150 x g for 10 minutes to obtain platelet-rich plasma (PRP). After additional centrifugation at 2000 x g for 10 minutes platelet-poor plasma (PPP) was obtained. By adding homologous PPP, platelet concentration of PRP was adjusted to 2 x 10^5^ μL^-1^. After administration of 20 μmol L^-1^ adenosine diphosphate (ADP), per cent platelet aggregation was assessed with the turbodimetric method using a ChronologLumiaggregometer with Aggro-Link Software. Platelet aggregation measured 5 min after addition of ADPwas used to determine on-treatment platelet reactivity. HPR was defined as the highest quartile of measured platelet reactivity in the examined population as reported previously [9,13–15].

In the validation cohort platelet function was analysed by Multiple Electrode Aggretometry (MEA). Samples of whole blood anticoagulated with hirudin were collected after initial clopidogrel loading. Platelet function was assessed after stimulation with 6.4 μM ADP, by a new generation impedance aggregometer (Multiplate Analyzer, VerumDiagnostica GmbH, Munich, Germany). Platelet activity was reported as area under the curve aggregation units (AUC) as described previously. A good correlation between MEA und LTA was shown before[16].

### Follow-up

Patients were followed up by telephone interview 12 month after enrollment. Incidence of major cardiovascular events (MACE) including death, myocardial infarction and ischemic stroke were assessed by telephone interview. An acute myocardial infarction (AMI) was diagnosed by a rise and/or fall of cardiac biomarker values [cardiac troponin (cTn)] with at least one value above the 99th percentile upper reference limit and with at least one of the following: symptoms of ischaemia, new or presumed new significant ST-segment–T-wave (ST–T) changes or new left bundle branch block, development of pathological Q waves in the ECG, imaging evidence of new loss of viable myocardium or new regional wall motion abnormality, or identification of an intracoronary thrombus by angiography [17].Telephone interviewers were blinded with respect to the results of platelet aggregation test.

### Statistical analysis

Continuous data with normal distribution are presented as mean ± SD, not normally distributed data as median and interquartile range. Categorical variables are expressed as number (%). Equality of distribution of categorical variables between subgroups was analyzed by chi-squared test. Continuous data with non-normal distribution were compared by Mann-Whitney test. For analysis of clinical predictors for HPR univariate logistic regression analysis was used. The highest quartile of on-treatment platelet reactivity assessed by LTA was used as the dependent variable. Clinical variables available in daily routine were included in the model as independent variables. Variables included age, gender, diabetes mellitus, hypertension, hyperlipidemia, smoking history, adiposity (BMI>30), reduced left ventricular function, reduced renal function and multivessel disease. For continuous variables cut-off values with highest sensitivity and specificity were determined by receiver operating characteristic (ROC) curves. Thus categorical variables were created that were used for development of the risk score. Factors with a significance level of P<0.1 in univariate analysis were included into multivariate model. Multivariate analysis was used then to identify independent predictors of HPR and to create the PREDICT-STABLE score. According to effect size (odds ratio) of relevant clinical predictors for HPR in multivariate analysis, a weighed score was developed. For comparison of MACE between different score levels, Kaplan-Meier curves were constructed and groups were compared by the log-rank test. For comparison of categorical and continuous data a two-sided P value of <0.05 was considered statistically significant. All statistical tests were performed with IBM SPSS Statistics software, version 21.0.

## Results

### Baseline characteristics

2226 patients were consecutively enrolled in the study. In 1549 patients platelet reactivity measurement by LTA was available, from these patients 810 (52.3%) were treated for acute coronary syndrome (unstable angina pectoris, non-ST- and ST-elevation myocardial infarction) and 739 (47.7%) for stable CAD, the latter were included in the analysis. Baseline patients’ characteristics are shown in Table 1. 30% of patients suffered from diabetes, 42.1% had reduced left ventricular function, 74.1% had multivessel disease. 65.7% were treated with bare metal stents, 24.5% with drug-eluting stents, 9.7% with both stent-types. Characteristics of study population according to quartiles of platelet reactivity are shown in Table 1. Patients in the highest quartile were significantly older, had a higher body mass index, had more often diabetes mellitus and renal impairment (P<0.05).

**Table 1:** Characteristics of the study population according to quartile of HPR.

Baseline demographics	Patients N = 739	Quartile of platelet reactivity				P*
		1	2	3	4	
Gender m/f (%)	77.5/22.5	75.5/24.5	80.7/19.3	81/19	72.8/27.2	0.081
Age (years)	69 (61–75)	67.0 (57–74)	68.0 (59.0–74.0)	70.0 (63.0–74.0)	69.0 (64.5–74.5)	0.051
Body mass index	27.4 (25.1–30.5)	26.1 (24.3–26.1)	27.1 (25.46–30.1)	28.4 (25.8–30.75)	28.3 (26.04–31.77)	0.001
Adiposity BMI>30 (%)	24.5	18.7	25.3	32.7	36.8	0.007
Hypertension (%)	82.9	80.1	81.8	86.4	87.6	0.129
Smoking history	39.0	42.4	40.8	38.9	38.1	0.536
Hyperlipidemia (%)	73.9	73.7	67.4	80.4	74.0	0.969
Diabetes mellitus (%)	30.9	22.6	27.1	34.2	41.0	0.001
Serum creatinin mg/dL	1.0 (0.9–1.2)	1.1 (0.9–1.2)	1.1 (0.9–1.3)	1.0 (0.9–1.28)	1.1 (0.9–1.4)	0.021
Left ventricular function (%)	55.7 (49–65)	55.7 (50–58)	60 (49–65)	60 (49–65)	54.5 (46–65)	0.083
Medication						
Statins (%)	88.9	87.0	90.2	91.4	86.9	0.367
ACE- Inhibitors(%)	77.4	77.6	76.8	79.3	76.0	0.615
Angiotensin receptor blockers (%)	11.6	13.7	8.5	16.5	14.9	0.514
β-blockers (%)	91.0	93.8	89.6	90.9	89.6	0.501
Multivessel disease	74.1	69.7	74.7	85.2	76.9	0.424
Bare metal stents/ drug-eluting stents/both	65.7/24.5/9.7	66.7/22.6/10.7	61.3/27.5/11.3	63.6/27.2/9.3	71.6/20.6/7.7	0.08/0.20/0.33

* for quartile 4 vs. 1–3.

### Risk factors of HPR and development of the risk score

**Fig 1:**
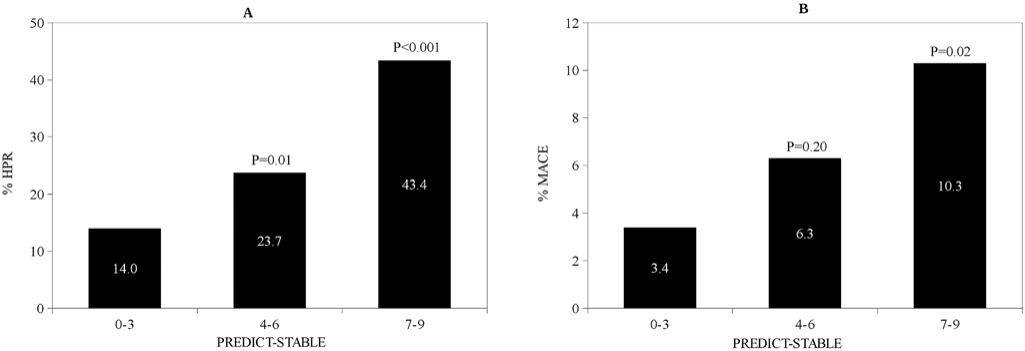
A Incidence of HPR (%) according to PREDICT-STABLE Score B Incidence of MACE according to PREDICT-STABLE Score. P-values for comparison with PREDICT-STABLE score 0–3

For continuous variables following cut offs with best sensitivity and specificity were calculated by ROC curves: age of 63 years, left ventricular function of 55% and serum creatinin level of 1.1 g/dL. These cut offs were used to create categorical variables for the analysis. In univariate analysis, the following factors were significant predictors of HPR (P<0.1, Table 2) and were included in multivariate analysis: age > 63 years, female gender, diabetes mellitus, adiposity (BMI >30 kg/m^2^), reduced left ventricular (LV) function (LV ejection fraction< 55%), reduced renal function (serum creatinin> 1.1 g/dL). In multivariate analysis age, diabetes mellitus, adiposity, reduced left ventricular function and reduced renal function remained significant predictors of HPR whereas female gender was no longer significant (Table 3). According to effect size (odds ratio) of relevant clinical predictors of HPR in multivariate analysis, a weighed score was developed. In detail age > 63 years was weighted by factor 3, diabetes and adiposity by factor 2, reduced left ventricular function and reduced renal function by factor 1 (odds ratios 2.11, 1.78, 1.86, 1.54 and 1.48 respectively). Thus, a score ranging from 0–9 was developed (Table 3). The prevalence for a PREDICT-STABLE score of 0–3 was 32.3%, 50% for a score of 4–6 and 17.7% for a score of 7–9. Fig. 1A shows per cent HPR in each score level: 14%, 23.7% and 43.4% for PREDICT-STABLE score 0–3, 4–6 and 7–9, respectively.

**Table 2:** Univariate analysis of risk predictors for HPR.

Quartile of platelet reactivity	1–3	4 (HPR)	Coefficient B	Odds ratio (95% CI)	P
Age (> 63 years) (%)	69.4	82.6	0.74	2.09 (1.37–3.20)	0.001
Gender m/f (%)	79.0/21.0	72.8/27.2	0.34	1.41 (0.96–2.06)	0.082
Diabetes mellitus (%)	27.9	41	0.58	1.79 (1.26–2.55)	0.001
Hypertension (%)	82.8	87.6	0.38	1.47 (0.89–2.42)	0.131
Hyperlipidemia (%)	73.9	74	0.01	1.01(0.69–1.48)	0.969
Smoking (%)	40.7	38.1	-0.11	0.54 (0.63–1.27)	0.536
Adiposity BMI>30 (%)	25.5	36.8	0.53	1.70 (1.16–2.51)	0.007
Reduced left ventricular function EF<55 (%)	41.5	51.4	0.39	1.48 (1.06–2.08)	0.02
Reduced renal function (Serumcreatinin> 1.1 g/dL in %)	46.5	61.5	0.61	1.84 (1.29–2.63)	0.001
Multivessel disease* (%)	74.9	71.9	-0.15	0.86 (0.59–1.25)	0.424

* defined as 50% or greater stenoses in at least one major epicardial vessel.

**Table 3:** Multivariate analysis of risk predictors for HPR.

Variables	Coefficient B	Odds ratio (95% CI)	P	PREDICT-STABLE
Age (> 63 years)	0.745	2.11 (1.26–3.53	0.005	3
Female gender	0.381	1.46 (0.93–2.31)	0.102	-
Diabetes mellitus	0.575	1.78 (1.19–2.65)	0.005	2
Adiposity (BMI>30)	0.622	1.86 (1.22–2.86)	0.004	2
Reduced left ventricular function EF<55	0.431	1.54 (1.03–2.31)	0.037	1
Reduced renal function (Serumcreatinin> 1.1 g/dL)	0.391	1.48 (0.97–2.25)	0.067	1

### Follow-up

**Fig 2:**
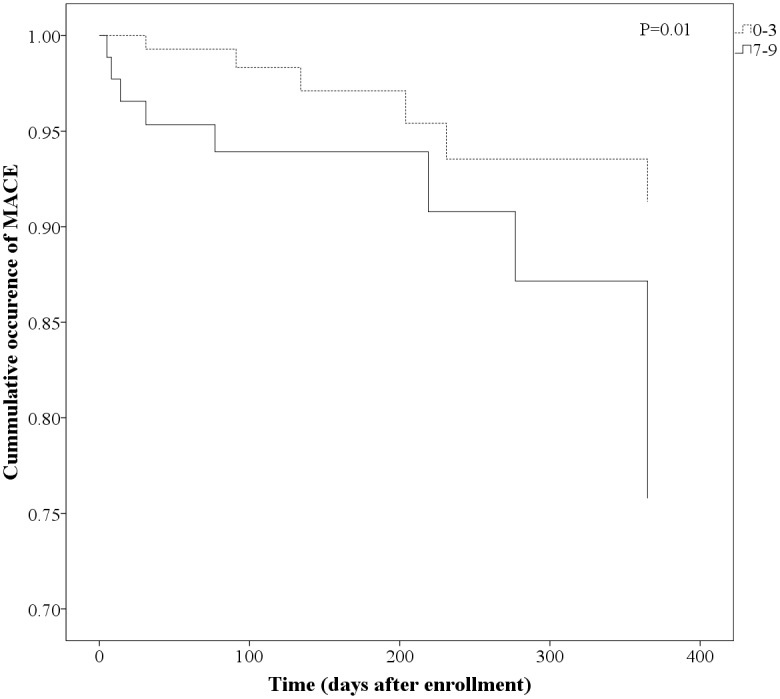
Kaplan-Meier analysis for incidence of MACE according PREDICT-STABLE Score (comparison of score levels 0–3 with 7–9).

**Fig 3:**
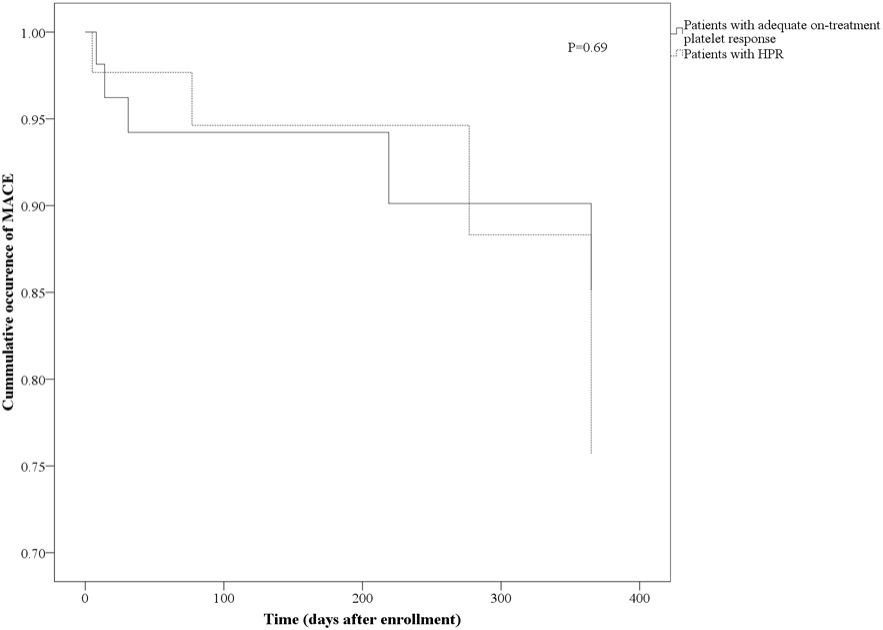
Kaplan-Meier analysis for incidence of MACE according platelet function analysis (comparison of HPR vs. adequate on-treatment response in Patients with high PREDICT-STABLE Score 7–9).

Follow-up was available for 686 patients (93%). MACE occurred in 42 (5.7%) of patients. Median follow up was 152 (91–400) days. There were 3.0% myocardial infarctions, 1.4% ischemic strokes and 1.9% deaths in the study population. Fig. 1B shows distribution of MACE according to PREDICT-STABLE score. There was a steady increase of MACE after 1 year follow up with higher PREDICT-STABLE score i.e. 3.4% in patients with a score of 0–3, 6.3% in patients with a score of 4–6 and 10.3% in patients with a score of 7–9 (P = 0.20 and 0.02 respectively for comparison with the score group 0–3). Patients with high PREDICT-STABLE score (7–9) had about 3 times higher probability to develop MACE than patients with score 0–3 (odds ratio 3.23, P = 0.02 for Score 7–9 vs. 0–3; odds ratio 1.90, P = 0.20 for Score 4–6 vs. 0–3). Early (30 days) and late outcomes are shown in Table 4. After 30 days there was numerically highest MACE rate for highest PREDICT STABLE score levels (7–9), although statistically not significant. Differences were consistent for all MACE components. Similarly, in Kaplan-Meier analysis a higher PREDICT-STABLE score level was associated with significantly higher MACE rate in comparison to a low score (Fig. 2, P = 0.01), however there was no difference in MACE between patients with HPR and patients with adequate on-treatment platelet reactivity (Fig. 3, P = 0.69).

**Table 4:** Clinical outcome after 30 days and 1 year of follow up according to PREDICT-STABLE Score.

30 days/1 year	N = 686	PREDICT-STABLE 0–3	PREDICT-STABLE 4–6	PREDICT-STABLE 7–9
MACE	1.8/5.7%	0.0/3.4%	1.4/6.3%	3.1/10.3%*
Myocardialinfarction	0.7/3.0%	0.0/1.7%	0.7/3.5%	1.0/5.2%
Ischemic stroke	0.3/1.4%	0.0/0.6%	0.4/1.8%	0.0/3.1%
Death	0.8/1.9%	0.0/1.1%	0.4/1.4%	2.1/4.1%
Stent thrombosis				
Definite	0.4/0.5%	0.0/0.6%	0.0/0.0%	3.1/3.1%
Probable	0.5/2.3%	0.0/0.6%	1.1/3.2%	0.0/3.1%
Possible	0.1/0.8%	0.0/0.6%	0.0/0.0%	0.1/2.1%

* P<0.05 in comparison with PREDICT-STABLE 0–3.

**Fig 4:**
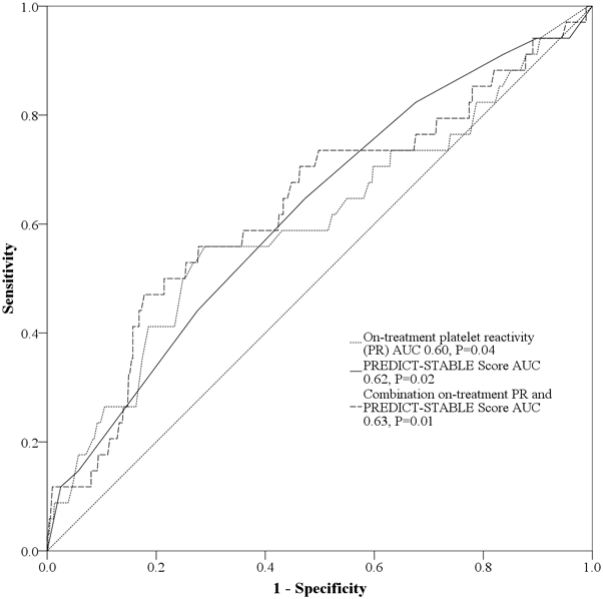
Comparison of predictive value for on-treatment platelet reactivity (PR), PREDICT-STABLE Score alone and in combination by ROC curve analysis.

ROC curve analysis showed that PREDICT-STABLE score improved prediction of 12-month MACE compared to on-treatment platelet reactivity alone as measured by the area under the curve (AUC 0.62, P = 0.02 versus 0.60, P = 0.04). However, there was no relevant benefit for risk prediction by combining both on-treatment platelet reactivity and PREDICT-STABLE score (AUC 0.63, P = 0.01, Fig. 4).

### Validation of the PREDICT-STABLE score in an independent cohort

**Fig 5:**
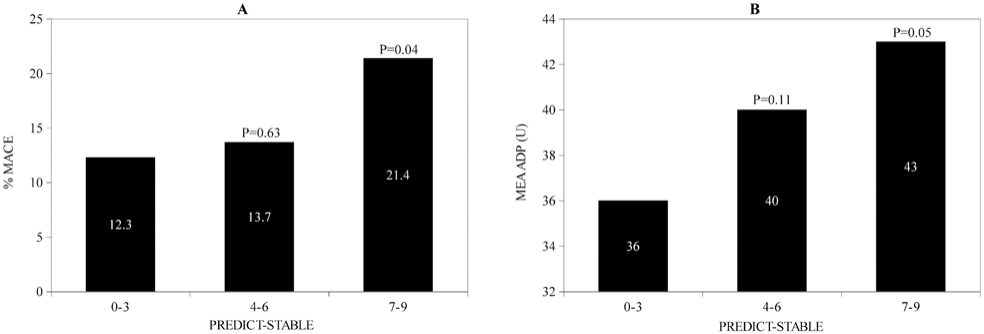
A Incidence of MACE according to PREDICT-STABLE Score in the validation cohort B Platelet reactivity assessed by MEA according to PREDICT-STABLE Score in the validation cohort. P-values for comparison with PREDICT-STABLE score 0–3.

For validation of the PREDICT-STABLE score 591 patients with same inclusion criteria as the study population were enrolled (Table 5). Similar to the exploratory cohort we could show a significant correlation of MACE with higher PREDICT-STABLE score levels (Fig. 5A). Furthermore, we could show that patients with higher PREDICT-STABLE score have significant higher platelet reactivity assessed by MEA (Fig. 5B). In analogy to the discovery cohort, combination between on-treatment platelet reactivity assessed by MEA and the PREDICT-STABLE score did not improve risk prediction for MACE in ROC-analysis.

**Table 5:** Baseline characteristics of the validation cohort.

Baseline demographics	N = 591
Gender m/f (%)	75/25
Age (years)	68 (59–76)
Adiposity BMI>30 (%)	28.4
Hypertension (%)	81.5
Smoking history	31.1
Hyperlipidemia (%)	69.1
Diabetes mellitus (%)	35.3
Reduced renal function (Serumcreatinin> 1.1 g/dL in %)	33.0
Reduced left ventricular function (%)	34.5
Medication	
Statins (%)	66.8
CE-Inhibitors or ARBs (%)	53.4
β-blockers (%)	67.2

## Discussion

Variability of individual response to antiplatelet therapy remains a challenging clinical problem. There is still a significant number of patientsafter elective coronary PCI having complicating adverse cardiovascular events [18–21]. This may be partly explained by variability of response to antiplatelet therapy. According to current data there are about 40% of patients considered to be low responders to clopidogrel depending on definition and particular platelet function assay [6,7]. The prognostic impact of high on-treatment platelet reactivity (HPR) for the occurrence of serious cardiovascular events after PCI, particularly stent thrombosis has been demonstrated in several studies [12–14,22,23]. However, there is currently no evidence that platelet function guided approaches lead to improvement of clinical outcome. To date, randomized trials investigating the effect of antiplatelet therapy adjusted to the results of platelet function testing have not shown an effect regarding improvement of outcome (GRAVITAS, TRIGGER-PCI, ARCTIC trial) either due to a weak active control arm (GRAVITAS) [6] or an overall low risk population (TRIGGER-PCI, ARCTIC) [7,8]. Therefore, the characterization of platelet function testing as a modifiable risk factor remains questionable. An association between genetic and non-genetic factors and HPR under dual antiplatelet therapy has been described previously [24–35]. Thus, it is in the focus of the on-going debate whether HPR is a “by-stander” of the overall cardiovascular risk rather than representing an independent modifiable parameter associated with clinical prognosis. Risk after elective non-urgent PCI is generally low. Some risk tools have been evaluated to characterize peri-procedural/in-hospital risk in stable CAD patients [36,37]. However, these scores have not been evaluated for their association with on-treatment platelet reactivity and long-term risk after elective PCI.

In own preliminary work we developed a score (PREDICT score) to estimate the likehood for HPR utilizing easily available non-genetic risk factors in an unselected cohort of patients with symptomatic coronary artery disease (stable CAD/ACS) [9]. The score contains distinct and easily available patient factors. In the present analysis, we were able to develop a modified score (PREDICT-STABLE Score) focusing on a sub-population of patients with stable CAD in a large retrospective cohort of patients. In our study age, adiposity defined by elevated BMI >30 kg/m2, diabetes mellitus, reduced renal function and reduced left ventricular function were associated with HPR. These findings are in line with previous studies. Influence of higher age on clopidogrel response could be demonstrated in several trials. As an explanation reduced liver function and hence slower activation of clopidogrel prodrug and higher baseline platelet reactivity are discussed [30,31]. In obese patients drug underdosing and decreased CYP3A4 activity is a possible explanation for lower response [32–34]. Enhanced vascular inflammation and platelet activation due to hyperglycemia, impaired lipid metabolism and oxidative stress can cause higher on-treatment platelet reactivity on antiplatelet treatment in diabetic individuals [26,27]. Some studies could demonstrate influence of reduced renal function on clopidogrel responsiveness. Altered platelet function and reduced sensitivity to antiplatelet drugs through complex disturbances (increased platelet turnover rate, impaired absorption or drug metabolism, procoagulant factors) in renal insufficient patients are supposed mechanisms [28,29,38,39]. In this context, individuals with moderate renal impairment might rather benefit from more intensified longterm P2Y12 inhibition [40]. Patients with heart failure show decreased gastrointestinal absorption of antiplatelet drugs, elevated markers of platelet activation including thromboglobulin, Platelet Factor 4, P-Selectin and platelet-derived adhesion molecules and increased platelet volume [35,41].

With the present analysis, we demonstrate that patients with a high PREDICT-STABLE Score (e.g. > 6 points) have significantly increased probability of HPR and higher rate of major adverse cardiovascular events. Moreover we could validate these findings in a separate consecutive cohort of patients with stable CAD. Of note, patients with a score of > 6 developed similar 12-month rate for atherothrombotic events as in clopidogrel-treated arms of recent major ACS-trials (Fig. 1B) [42,43]. Additionally, we did not find any improvement of prediction by adding on-treatment platelet reactivity to assessment of clinical risk factors for HPR and atherothrombotic events. This adds to the hypothesis that HPR represents more a bystander of overall atherothrombotic risk and partly explains the previous unsuccessful approaches to use platelet reactivity as a modifiable risk factor alone. This is especially true for long-term prognosis after PCI in stable CAD patients in contrast to the ACS setting when there is a biologically causative relationship between high platelet reactivity and early atherothrombotic events including stent thrombosis. In line with these observations are previous results from studies including stable CAD patients and medically managed ACS patients revealing that HPR has no incremental benefit for risk prediction over established clinical risk factors for MACE [44,45]. Hence, additional clinical risk factor assessment rather than isolated platelet function-guided approaches should be investigated in future to evaluate personalized antiplatelet strategies in stable patients with coronary artery disease. It is tempting to speculate, that the score might help to identify patients at high risk (e.g. patients with high PREDICT-STABLE score) that benefit not only from more intensified antiplatelet regimen (ticagrelor, prasugrel, low dose rivaroxaban or vorapaxar) [42,43,46,47] but also from a more universal strategy to modify risk profile thus reducing atherothrombotic events. Interventional studies are needed to characterize the effects of these approaches to demonstrate effects on thromboischemic and bleeding risk in selected high risk stable CAD patients.

### Limitations

Antiplatelet drug response is multifactorial. In our score we assessed clinical risk variables which can be easily obtained from patients’ clinical examination. Although LTA is still the gold-standard to investigate ADP-induced aggregation, antiplatelet drug response is more complex than monitored by a single platelet function test. Additionally, several genetic factors affect response to clopidogrel (polymorphisms of CYP3A4, CYP2C19, GPIa, P2Y12, GPIIIa, CES1) and these were not included in the present study. There can be more unknown factors which were not considered in the present score. On the contrary, avoiding extensive laboratory testing makes the score valuable for application in clinical situations when rapid information and risk assessment is needed.
